# Supplementary material for: Do Bryophyte Elemental Concentrations Explain Their Morphological Traits?
Source: Plants (Basel). 2021 Jul 31;10(8):1581. doi: 10.3390/plants10081581 (PMC8398013; doi:10.3390/plants10081581)
Supplement: Supplementary file 1 [file plants-10-01581-s001.zip › plants-1314011-supplementary.pdf]

plants

MDPI

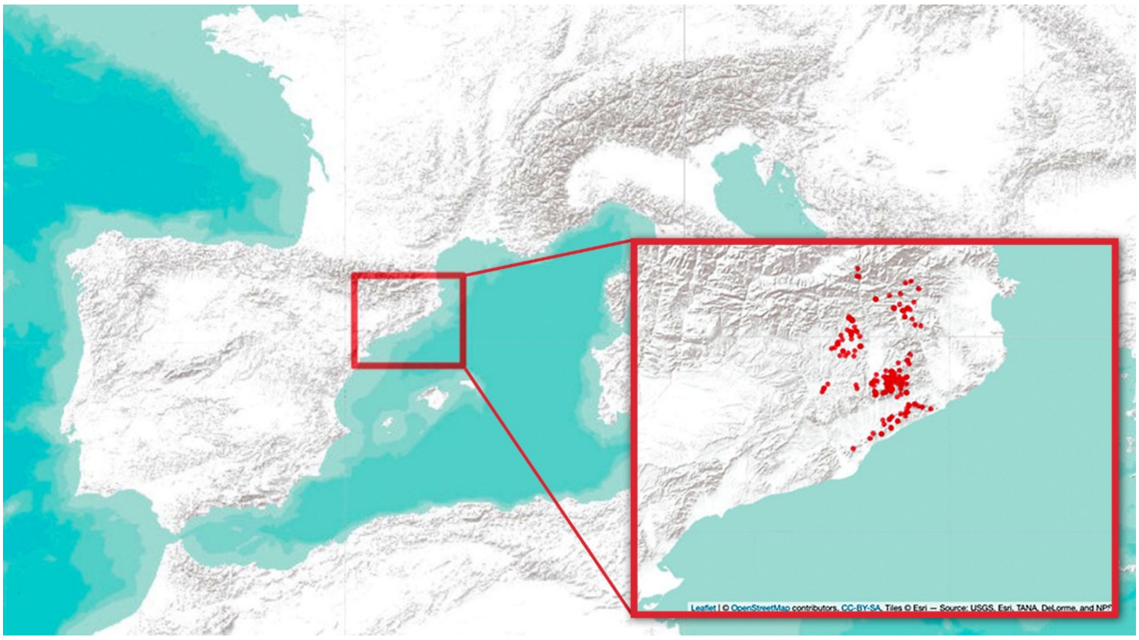

Figure S1. Map showing the location of the sampled bryophytes (red dots).

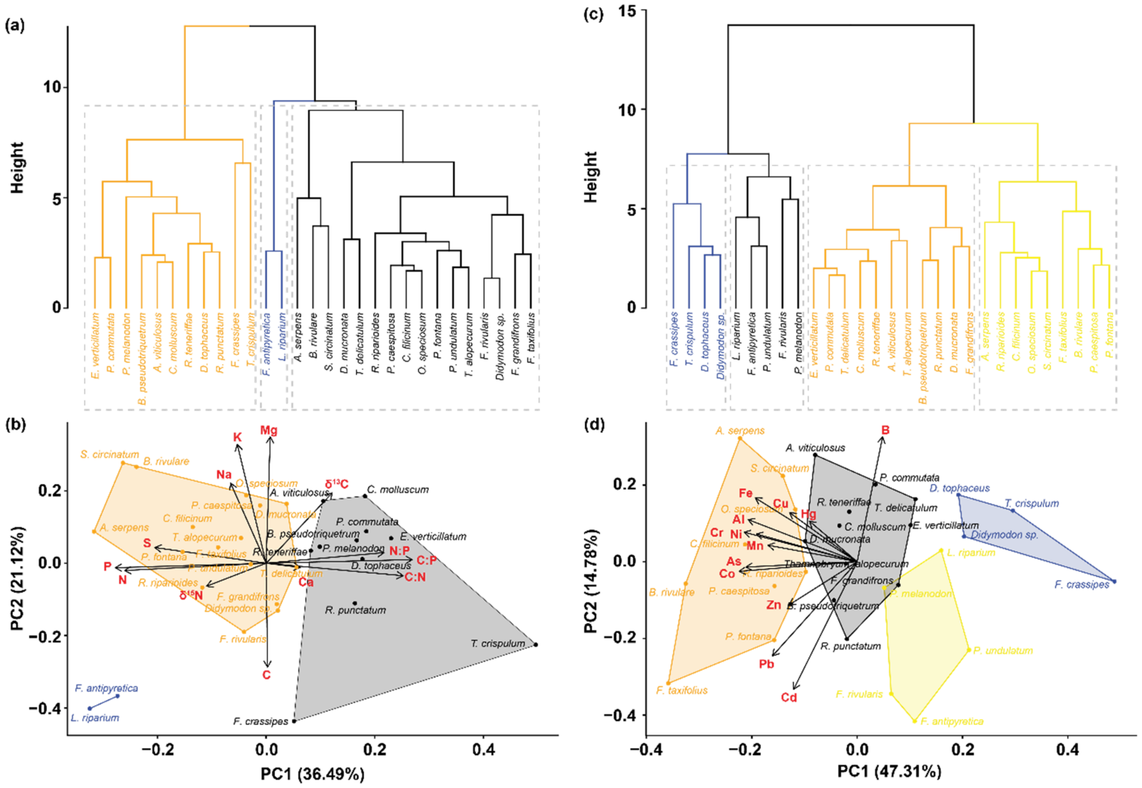

Figure S2. Cluster and PCA analyses showing the aggregation of different species in terms of their C:N:P stoichiometry and other macroelements (**a**, **b**) and trace and heavy metals (**c**, **d**).
